# Negative Regulators of JAK/STAT Signaling in Rheumatoid Arthritis and Osteoarthritis

**DOI:** 10.3390/ijms18030484

**Published:** 2017-02-24

**Authors:** Charles J. Malemud

**Affiliations:** Department of Medicine, Division of Rheumatic Diseases, Case Western Reserve University School of Medicine, University Hospitals Cleveland Medical Center, Foley Medical Building, 2061 Cornell Road, Room 207, Cleveland, OH 44106-5076, USA; cjm4@cwru.edu; Tel.: +1-216-844-7846 or +1-216-536-1945; Fax: +1-216-844-2288

**Keywords:** arthritis, apoptosis, chondrocytes

## Abstract

Elevated levels of pro-inflammatory cytokines are generally thought to be responsible for driving the progression of synovial joint inflammation in rheumatoid arthritis (RA) and osteoarthritis (OA). These cytokines activate several signal transduction pathways, including the Janus kinase/Signal Transducers and Activators of Transcription (JAK/STAT), Stress-Activated/Mitogen-Activated Protein Kinase (SAPK/MAPK) and phosphatidylinositol-3-kinase/Akt/mechanistic target of rapamycin (PI3K/Akt/mTOR) pathways which regulate numerous cellular responses. However, cytokine gene expression, matrix metalloproteinase gene expression and aberrant immune cell and synoviocyte survival via reduced apoptosis are most critical in the context of inflammation characteristic of RA and OA. Negative regulation of JAK/STAT signaling is controlled by Suppressor of Cytokine Signaling (SOCS) proteins. SOCS is produced at lower levels in RA and OA. In addition, gaining further insight into the role played in RA and OA pathology by the inhibitors of the apoptosis protein family, cellular inhibitor of apoptosis protein-1, -2 (c-IAP1, c-IAP2), X (cross)-linked inhibitor of apoptosis protein (XIAP), protein inhibitor of activated STAT (PIAS), and survivin (human) as well as SOCS appears to be a worthy endeavor going forward.

## 1. Introduction

Numerous pro-inflammatory cytokines including, interleukin-1β (IL-1β), IL-2, IL-3, IL-6, IL-7, IL-8, IL-12/23, IL-17, IL-18, IL-19/IL-20, IL-32, IL-35, tumor necrosis factor-α (TNF-α), interferon-α/γ (IFNα/γ) and oncostatin M (OSM) are prominently elevated in rheumatoid arthritis (RA) where they are involved in immune-mediated inflammation characteristic of this autoimmune disease [1,2,3,4,5]. Three of these cytokines in particular, namely IL-1β and IL-6 and TNF-α, were also found at elevated levels in patients diagnosed with osteoarthritis (OA) [6,7,8]. 

A common thread which appears to link these pro-inflammatory cytokines to inflammation is their capacity to activate the Janus Kinase/Signal Transducers and Activators of Transcription (JAK/STAT) signaling pathway [9,10,11,12]. In that regard, several downstream cellular events known to be dependent on the phosphorylation (i.e., activation) of STAT proteins were also found to be responsible for perpetuating a state of chronic inflammation in autoimmune diseases [13,14,15] as well as being crucial for regulating cell survival, controlled cell death (apoptosis), necropotosis, differentiation and fate determination [12]. It was also reported that the level of three anti-inflammatory cytokines, notably IL-4, -5 and -13, produced by the T_H_2 T-cell subtype (as measured by expression of the transcription factor, GATA-3) would be dampened in RA [16]. Thus, when the results of these studies were considered together, they firmly established that the ratio of pro-inflammatory to anti-inflammatory cytokines in RA was markedly skewed towards the former. 

The conceptual framework established by basic research and clinical studies was that the phosphorylation of STAT proteins (i.e., p-STAT) would also be dampened and concomitantly those molecular events that promote and perpetuate chronic inflammation would be ameliorated by neutralizing the interaction between pro-inflammatory cytokines and their respective receptors. In that respect, the development of tocilizumab, a monoclonal antibody which inhibits the interaction between IL-6 and the various forms of the IL-6 receptor, was the perceived underlying mechanism for neutralizing IL-6 activation of the JAK/STAT signaling pathway in RA [17,18,19,20]. 

The level of TNF-α is significantly elevated in the sera and synovial fluid of both RA and OA patients. Thus, accumulating evidence has suggested the distinct possibility that TNF-α and IL-1β are capable, at least in vitro, of activating the JAK/STAT pathway through which the frequency of apoptotic chondrocytes could be increased. In fact, based on these findings, using the TNF-α or IL-6 receptor blockade to achieve clinical remission in active RA patients would be predicted to shut down STAT protein activation even though blockade of TNF-α or IL-6 would also likely involve their more conventional site of action on signal transduction, namely the Stress-Activated/Mitogen-Activated Protein Kinase (SAPK/MAPK) pathway [21,22,23]. 

Activation of JAK/STAT by pro-inflammatory cytokines is now considered to be of major importance in driving chronic inflammation in RA [24]. This advance took on additional clinical significance which eventually resulted in the development of two JAK-selective small molecule inhibitors (SMIs), namely tofacitinib (JAK3) and ruxolitinib (JAK1/JAK2) [25,26,27,28], and especially tofacitinib, which has become part of the overall RA drug armamentarium. In contrast, ruxolitinib has been primarily employed for treating myeloproliferative neoplasms and psoriasis [29]. 

However, another possibility that could account for the deregulation of JAK/STAT signaling in RA and OA must also be seriously considered. This refers to the impact of two main endogenous negative regulators of JAK/STAT, notably Suppressor of Cytokine Signaling (SOCS) [30] and cellular Inhibitor of Apoptosis Proteins (c-IAPs), including the Protein Inhibitor of Activated STAT (PIAS) [12,31]. 

## 2. Suppressor of Cytokine Signaling

### 2.1. Mechanisms Attributed to Activity of Suppressor of Cytokine Signaling 

Suppressor of Cytokine Signaling (SOCS) belongs to a class of potent endogenous negative regulators of JAK/STAT pathway signaling [12,32,33,34]. My recent review on this subject discussed the role of SOCS in RA which emphasized that SOCS activity was a critical feature for both the positive and negative regulation of macrophage and dendritic cell activation as well as being important for the capacity of SOCS to immunomodulate inflammatory responses associated with autoimmune diseases [30]. Thus, it was germane to examine the extent to which SOCS activity was limited in RA which would, in part, permit chronic inflammation to be perpetuated. 

At approximately the same time that constitutive STAT protein activation was discovered to be a mechanism associated with the aberrant survival of cells in several chronic disorders including various types of cancer [35,36,37] and human immunodeficiency virus (HIV)-1 [38], up-regulation of the SOCS protein class in these conditions was also identified, which was accompanied by the activation of JAK/STAT via the action of various cytokines. 

To summarize, several forms of SOCS have been characterized. This intracellular class of proteins is comprised of seven SOCS forms, namely SOCS-1 through SOCS-7, as well as the cytokine-inducible SH2 domain protein, termed CIS, now also known as CISH [12,30]. In early studies which examined the biological activity of SOCS proteins, El Kasmi et al. [14] found that by eliminating the binding of SOCS-3 to JAK3, the IL-6/IL-6Rα/gp130 pathway generated an anti-inflammatory response in cultured macrophages. Furthermore, in studies on the structure of SOCS proteins, Yoshimura et al. [33] showed that SOCS proteins were comprised of a centrally situated SH2 domain surrounded by an NH_2_-terminal domain of variable length at one end of the molecule and a carboxy-terminal 40-amino-acid domain known as the “SOCS box” at the other end, the latter identified as the structural entity which was critical for directing STAT proteins to the ubiquitin transferase–mediated degradation (i.e., proteasome) pathway. 

Several additional studies implicated SOCS as a critical negative regulator of STAT proteins which worked through another mechanism in addition to its role in directing proteasome-mediated degradation of STAT proteins. For example, Kershaw et al. [39] showed that SOCS3 binds to specific elements comprised of a receptor/JAK complex which regulates cytokine signaling by directly inhibiting the kinase. Another alternative mechanism besides proteasome-mediated degradation involved SOCS-mediated steric hindrance (reviewed in [12]). This mechanism ultimately results in altered JAK activation which had been previously shown to be required for the recruitment and activation of STAT proteins. In that regard, Giordanetto et al. [40] verified through an in-depth structural analysis of SOCS-1/JAK2 binding that SOCS-1 bound directly to JAK2 via its SH2 domain, whereas De Souza et al. [41] showed that the SOCS-3 SH2 domain bound directly to the cytokine receptor, thus limiting JAK-mediated STAT protein activation. Moreover, the SH2 domain and protein phosphatase SHP2, the latter another negative regulator of STAT activation [12], had similar binding specificities.

### 2.2. Suppressor of Cytokine Signaling and Rheumatoid Arthritis

The pathophysiology of RA characteristically involves several deregulated cellular events responsible for altered innate and adaptive immune responses which significantly impact the structural alterations observed in articular cartilage and subchondral bone in this disease [42,43]. My recent review, which focused on the role of SOCS in human RA [30], indicated that the resultant hyperplastic synovial tissue characteristic of RA is comprised of a host of activated immune cell types, including T- and B-lymphocytes as well as macrophages, dendritic cells, mast cells, neutrophils and fibroblast-like synoviocytes (FLS), all of which appear to be “apoptosis-resistant” [44], whereas the frequency of apoptotic chondrocytes is elevated, thus potentially reducing chondrocyte vitality and viability [30,44]. 

In contrast, the erosions of subchondral bone in RA joints are orchestrated by activated osteoclasts which are plentiful in RA pannus. The result is a skewing of the balance between limited bone formation/turnover and bone resorption towards the latter. Of note, the results of a study reported by Choe et al. [45] indicated that Receptor Activator of Nuclear Factor-κB Ligand (RANKL) expression, which in RA contributes to the elevated level of osteoclast differentiation, is regulated by the IL-6/soluble IL-6 receptor, the JAK2/STAT3/SOCS-3 pathway. Furthermore, this group showed that the calcineurin inhibitor, tacrolimus, inhibited RANKL expression in RA-FLS by suppressing STAT3, but most importantly SOCS-3 was also induced by tacrolimus. Taken together, these results suggested that reduced SOCS-3 levels in RA may be, in part, responsible for initiating subchondral bone erosions. 

It is now well accepted that pro-inflammatory cytokines which activate the JAK/STAT pathway as well as the transcription factor Nuclear Factor Kappa B (NF-κB) [46] are critically involved in perpetuating a state of chronic inflammation which can be traced to the pathophysiological events in RA that are described above. However, having recently reviewed the role of SOCS in RA [30] we also concluded that negative regulation of STAT protein activation via SOCS was also deficient. In addition, I recently pointed out to the best of my knowledge, which was also confirmed by a literature search using the PubMed database, that there have yet to be any RA clinical trials which have employed strategies designed to increase SOCS-based regulation of JAK/STAT signaling, although Shouda et al. [47] had predicted that over-expressing SOCS3 in RA synovial tissue might have clinical benefit by dampening the clinical symptoms of RA. In that regard, a recent review by Mahony et al. [48] proposed that a vigorous commitment to focus on SOCS-3 as a therapeutic target in RA should now be considered.

### 2.3. Suppressor of Cytokine Signaling and and Osteoarthritis

It is now recognized that there is a “final common pathway” that links the progression of RA and OA pathophysiological alterations in articular cartilage [49]. These pathways include those that regulate chronic inflammation, the increased frequency of apoptotic chondrocytes, and, most critically, the up-regulation of matrix metalloproteinase (MMP) gene expression. These pathways are also controlled by a significant up-regulation of pro-inflammatory cytokine gene expression which has been shown to be regulated by activation of the JAK/STAT pathway [12]. For example, Lim and Kim [50] showed that MMP-13 (i.e., collagenase-3) expression in an IL-1β–treated human chondrosarcoma cell line (employed as a surrogate for human chondrocytes) induced JAK2 and STAT-1/STAT-2 activation as well as MMP-13 gene expression which was blocked by the *pan*-tyrosine kinase inhibitor AG490. However, treating this chondrocyte cell line with IL-1β also activated p38-MAPK/c-Fos/activator protein-1 (AP-1), which we had previously shown to be the case with human chondrocytes derived from OA cartilage [51]. In another study, de Andrés et al. [52] showed that levels of SOCS were lower in human OA chondrocytes compared to chondrocytes from non-arthritic cartilage samples. Specifically, SOCS-2 and CIS-1 mRNA levels were 10-fold lower compared to normal chondrocytes. However, SOCS-1 and SOCS-3 mRNA levels were comparable. Of note, SOCS-2 and CIS-1 mRNA levels were reduced six-fold and three-fold, respectively, when chondrocytes were incubated with IL-1β and OSM, in combination with TNF-α, suggesting that the pro-inflammatory cytokines implicated in OA progression and altered cartilage extracellular matrix structure were likely associated with activated JAK/STAT via the reduced expression of the SOCS-2 and CIS-1 genes. Another aspect of this study which may have significance for future directions was the finding that reduced levels of SOCS-2 were not associated with altered CpG methylation patterns in the SOCS-2 promoter and, moreover, that cytokine stimulation did not alter this pattern. To summarize, although follow-up studies have not apparently confirmed or refuted these findings, it appears reasonable to conclude at this time that the SOCS-mediated negative regulation of JAK/STAT signaling is deficient in OA. 

## 3. Cellular Inhibitor of Apoptosis Protein-1 (c-IAPs) as Inhibitors of Apoptosis: Potential Effect on JAK/STAT Signaling

### Role of Cellular Inhibitor of Apoptosis Protein-1 (c-IAPs) in Apoptosis 

PIAS belong to the c-IAPs class of proteins, which regulate the overall frequency of apoptosis during normal homeostasis, including cell survival and tissue turnover [12,15,20]. In addition to the five members of the PIAS protein group, PIAS1, PIAS2, PIASx, PIAS3, PIAS4, also known as PIASy [53], the group of c-IAPs also include apoptosis signal-regulating kinases [31], various cytokines [34] and p53 up-regulated modulator of apoptosis (PUMA) [34,54]. This class of proteins became a target for therapeutic intervention when it was shown that they were frequently deregulated in cancer [55]. However, importantly, in the present context, the c-IAPs might also be potential targets for intervention in autoimmune diseases such as RA, and perhaps also in OA. 

Apoptosis is regulated by a balance between pro-apoptotic and anti-apoptotic proteins. In that regard, the principal function of c-IAPs (i.e., c-IAP1, c-IAP2) is to ubiquitylate receptor-interacting serine/threonine-protein kinase 1 (RIPK1) [56], comprising the death receptor signaling complex, resulting in the activation of NF-κB [57]. This process involving RIPK1 (and RIPK3) is referred to as necroptosis because it involves the rupture of cells ‘triggered’ by TNF, Toll-like receptors or the T-cell receptor when pro-apoptotic caspase-8 is inhibited [58,59]. Therefore, when caspase-8 activity is low or absent, RIPK1 associates with RIPK3 which “triggers” necroptosis by RIPK3-mediated phosphorylation of pseudo-kinase MLKL—mixed lineage kinase ligand [60]. Furthermore, evidence favors the deubiquitination of RIPK1 as a mechanism resulting in the loss of IAP activity and the activation of deubiquitinase [61]. 

To summarize, c-IAPs and PIAS in particular can negatively regulate apoptosis through their ability to (1) inhibit caspase activity; (2) neutralize the activation of pro-caspases; and (3) by acting as ubiquitin-ligases which results in proteasomal degradation of pro-apoptotic proteins as well as transcription factors, such as activated STAT proteins, which control, in part, the expression of cytokines and apoptosis-related proteins (also reviewed in [12]). The role of protein-protein interactions including the formation of protein–c-IAP complexes would also provide an alternative mechanism for regulating apoptosis, which was reviewed by Lewis and Malemud [62]. For example, in the context of RA, c-IAPs were shown to be functionally involved in TNF-α–induced apoptosis which was traced to the finding that X (cross)-linked inhibitor of apoptosis (XIAP) interacted with mitogen-activated kinase kinase kinase 2 (MEKK2) [55] to alter NF-κB activation. Thus, it is highly likely that the significance of “apoptosis resistance” in RA synovium and the increased frequency of apoptotic chondrocytes in RA and OA result from changes in the activity of XIAP. In fact, this proved to be the case with second mitochondria-derived activator of caspase (Smac) [63]/direct inhibitor of apoptosis binding protein with low isoelectric point (DIABLO) [64], referred to as Smac/DIABLO, an inhibitor of XIAP activity. In that context, DIABLO may act to promote apoptosis through its capacity to bind to IAPs which, in turn, would prevent IAPs from inhibiting caspases. 

In the present discussion on the role of XIAP in arthritis, an early key finding was provided by Dharmapatni et al. [65] who showed that the level of XIAP and another anti-apoptosis factor, survivin, were significantly elevated in synovial tissue from patients with active RA. Importantly, even high levels of tumor necrosis factor-related apoptosis-inducing ligand (TRAIL) were not sufficient enough to overcome elevated levels of XIAP and survivin. In that respect, several strategies were employed which were designed to decrease XIAP levels as a potential therapy for cancer (reviewed in [62]). More recently, Dharmapatni et al. [66] found that low-dose embelin (2,5-dihydroxy-3-undecyl-1,4-benzoquinone), an inhibitor of XIAP, suppressed inflammation and bone erosion in the collagen antibody-induced (CAIA) model of arthritis in mice. Low-dose embelin also reduced the level of carboxy-terminal collagen crosslinks without reducing XIAP expression. Importantly, treatment of CAIA mice with low-dose embelin also increased the number of terminal deoxynucleotidyl transferase dUTP nick end labeling (TUNEL)-positive cells. However, a dissociation may exist between the suppression of inflammation and cell death. For example, Lawlor et al. [67] showed that RIPK3 was capable of promoting NLR family pyrin domain containing 3 (NLRP3) inflammasome and IL-1β responses independent of mixed-lineage kinase (MLK) domain-like, and necropototic cell death. In the same manner, aberrant IL-1β secretion from dendritic cells was found to be dependent on TNF and RIPK3 but independent of c-IAP1/c-IAP2 [68].

Lewis and Malemud [62] discussed the potential role of embelin and an embelin derivative known as 6g with improved binding to the BIR3 domain of XIAP (6 g: *K*i = 0.18 ± 0.09 μM compared to *K*i = 0.40 ± 0.13 μM for the parent compound). Thus, embelin may be exploited as a potential inducer of apoptosis. Mechanistic target of rapamycin (mTOR), a phosphoinostide-3-kinase protein kinase, may also play a role as a regulator of T-cell homeostasis [69]. 

Recently, Lao et al. [53] showed that RA-FLS as well as RA synovial tissue expressed increased levels of PIAS3, but not PIAS1, PIAS2 or PIAS4. A further evaluation indicated that PIAS3-mediated SUMOylation of Rac1/PAK1 regulated the downstream activity of p21-activated kinase (PAK1) and c-Jun terminal-amino kinase (JNK). Of note, inhibition of the small GTPase Rac1, PAK1 or JNK inhibited the migration and invasion of RA-FLS, suggesting that in RA, suppression of PIAS3 may be protective against further synovial joint destruction. However, at the time that this manuscript was being prepared for publication, a search of the PubMed database did not reveal any published studies pertaining to the direct effect of suppressing PIAS on JAK/STAT signaling in RA or the extent to which PIAS was altered in either OA synovial tissue, subchondral bone or articular cartilage.

## 4. Conclusions and Future Perspectives

Pro-inflammatory cytokine–induced activation of JAK/STAT signaling appears to be critical for perpetuating the chronic state of inflammation characteristic of the progression of RA and OA to synovial joint failure. One current state of research in this area focuses on unraveling the molecular mechanisms which regulate aberrant cell survival, apoptosis and matrix metalloproteinase gene activity in these conditions. Additionally, the negative feedback loop that regulates JAK/STAT signaling via SOCS and c-IAPs appears to be altered in RA. This deregulation of JAK/STAT signaling results in the aberrant survival of activated immune cells and synoviocytes coupled to the failure of these cells to undergo apoptosis and may constitute a working hypothesis for investigating why chondrocyte apoptosis is increased in OA cartilage. Thus, future therapies for cancer, RA (and perhaps even OA) may eventually arise from a concerted effort to discover novel targets that will improve the capacity of SOCS and/or c-IAPs to restore normal JAK/STAT signaling [70,71,72]. This could result in increased apoptosis or necropotosis in cancer cells and synovial tissue, suppression of apoptosis in RA and OA articular cartilage, as well as blunting of subchondral bone alterations in both musculoskeletal conditions. 

## Abbreviations

Akt	Ak refers to a mouse strain which developed spontaneous thymic (t) lymphoma
AP-1	activator protein-1
c-Fos	a member of the Fos family of oncogenes
MEKK2	mitogen-activated kinase kinase kinase 2
MLK	mixed lineage kinase
MLKL	mixed lineage kinase ligand
mTor	mechanistic target of rapamycin
NLRP3	NLR family pyrin domain containing 3
TUNEL	terminal deoxynucleotidyl transferase dUTP nick end labeling
